# Heritability of Sleep EEG Topography in Adolescence: Results from a Longitudinal Twin Study

**DOI:** 10.1038/s41598-018-25590-7

**Published:** 2018-05-09

**Authors:** Andjela Markovic, Peter Achermann, Thomas Rusterholz, Leila Tarokh

**Affiliations:** 1 University Hospital of Child and Adolescent Psychiatry and Psychotherapy, University of Bern, Bern, Switzerland; 2 Institute of Pharmacology and Toxicology, University of Zurich, Zurich, Switzerland; 3 Neuroscience Center Zurich, University of Zurich and ETH Zurich, Zurich, Switzerland; 4 Zurich Center for Integrative Human Physiology, University of Zurich, Zurich, Switzerland; 5 Zurich Center for Interdisciplinary Sleep Research, University of Zurich, Zurich, Switzerland; 6 Department of Psychiatry and Human Behavior, Alpert Medical School of Brown University, Providence, RI USA

## Abstract

The topographic distribution of sleep EEG power is a reflection of brain structure and function. The goal of this study was to examine the degree to which genes contribute to sleep EEG topography during adolescence, a period of brain restructuring and maturation. We recorded high-density sleep EEG in monozygotic (MZ; n = 28) and dizygotic (DZ; n = 22) adolescent twins (mean age = 13.2 ± 1.1 years) at two time points 6 months apart. The topographic distribution of normalized sleep EEG power was examined for the frequency bands delta (1–4.6 Hz) to gamma 2 (34.2–44 Hz) during NREM and REM sleep. We found highest heritability values in the beta band for NREM and REM sleep (0.44 ≤ h^2^ ≤ 0.57), while environmental factors shared amongst twin siblings accounted for the variance in the delta to sigma bands (0.59 ≤ c^2^ ≤ 0.83). Given that both genetic and environmental factors are reflected in sleep EEG topography, our results suggest that topography may provide a rich metric by which to understand brain function. Furthermore, the frequency specific parsing of the influence of genetic from environmental factors on topography suggests functionally distinct networks and reveals the mechanisms that shape these networks.

## Introduction

During sleep, a time when conscious perception is suspended, the brain generates stereotypic cortical oscillations unique to this behavioral state^1^. These oscillations can be readily measured by means of the electroencephalography (EEG), and reflect underlying neuroanatomy and neurophysiology. Two sleep specific oscillations that embody this principle are slow waves and sleep spindles. Slow waves are high-amplitude low frequency waves (<5 Hz) that reflect the depth of sleep and are primarily generated through cortico-cortical and thalamo-cortical loops^2^, while spindles are transient oscillations between 11 and 16 Hz and are generated through thalamo-cortical loops^3–7^.

The magnitude of sleep EEG oscillations shows topographic variation dependent on frequency band^8–12^. For example, activity associated with slow waves (slow wave activity; SWA; <5 Hz) during non-rapid eye movement sleep (NREMS) exhibits a maximum over frontal regions, whereas oscillations in the theta frequency range (4.8–8 Hz) during NREMS peak over occipital brain areas^8^. Sleep spindles, reflected in sigma power (11–16 Hz) during NREMS, have maximum power over a confined region over the vertex. These regional differences suggest that sleep is not a uniform phenomenon and possibly reflect the multiple generators underlying the sleep EEG signal.

Similar to the sleep EEG power spectrum, which is an established trait^13–16^, the topographic distribution of power may also be unique to an individual^17,18^. Finelli *et al*.^17^ examining multiple sleep EEG recordings in the same individuals found that the similarity between power maps from two nights of the same person was significantly higher than when comparing two non-related individuals. Thus, the sleep EEG may exhibit stable topography, potentially adding another dimension in which an individual subject can be identified.

Although the above study suggests intra-individual stability in adults, during development and aging^19^ the regional distribution of sleep EEG power exhibits significant age-related shifts. One longitudinal study examining SWA across adolescence at five EEG derivations found that the adolescent decline in power showed a posterior to anterior progression, occurring first at an occipital derivation and last at a frontal derivation^20^. Similarly, in their cross-sectional study, Kurth *et al*.^10^ examined changes in NREMS topography across development spanning ages 2.4 and 19.4 years using high-density (128 channel) EEG. Maximal power in SWA showed an age-related progression from posterior to anterior brain regions through childhood and adolescence. This same trajectory of posterior to anterior has been observed for cortical maturation in neuroimaging studies^21^, suggesting that SWA may reflect underlying cortical maturation. Further evidence for this notion comes from a study by Buchmann *et al*.^22^ in which sleep EEG SWA and grey matter volume as measured via magnetic resonance imaging were recorded in the same subjects.

Concurrent to the shift in the region with maximal SWA, a significant global decrease in EEG power has been shown during adolescence^10,23–33^. It has been proposed, that this decrease might reflect synaptic pruning, a process that is involved in adolescent brain development and includes elimination of unused synapses^23,34^. Taken together, these findings indicate that brain oscillations during sleep reflect the underlying processes involved in brain maturation.

Despite these developmental changes, trait-like features have been detected in the sleep EEG of healthy children and adolescents^13,14^. Tarokh *et al*.^14^ examined normalized sleep EEG power spectra in four EEG derivations from two consecutive nights at two longitudinal time points separated by several years and observed high within-subject stability and inter-individual variability which was in a similar range as those reported in adults^15,16^. Such trait-like features may reflect a genetic contribution to the sleep EEG. Twin studies are the most suitable design for quantifying the degree to which genes contribute to the sleep EEG. Their results are based on a comparison of sleep EEG power in monozygotic (MZ) twins, who share approximately 100 % of their genes, and dizygotic (DZ) twins, who only share about 50 % of their genetic material^35^. Therefore, greater similarity between monozygotic twins as compared to dizygotic twins can be attributed to the larger proportion of shared genes. Studies in adult twins have shown that the sleep EEG power spectrum is highly heritable^13,36–38^.

To our knowledge, only one twin study analyzed regional aspects of EEG power^39^. Gao *et al*.^39^ examined ***waking*** alpha power and frontal alpha asymmetry in 9–10 year old twins. They found high heritability of alpha power (70 – 85 % of the variance due to genes) and a modest but significant heritability in frontal alpha asymmetry (11–27 % of the variance due to genes). Despite the dearth of studies examining the genetic contribution to topography, topographic aspects of the EEG are of significance – aberrations in topographic distribution have been observed in several psychiatric^40–42^ and neurological^43–47^ disorders, emphasizing its importance. Furthermore, differences in sleep EEG power between patient populations and healthy controls are almost always over a circumscribed brain region and may be a result of the neural circuitry implicated in a specific disorder. For example, in children with attention deficit hyperactivity disorder, an increase in SWA in central regions is observed^41^, while in adolescents with major depressive disorder an increase of SWA over frontal regions is observed^48^. These studies imply that the topography of the sleep EEG may be an additional marker of functional brain circuitry.

Therefore, the primary aim of the current study was to examine heritability of sleep EEG topography using high-density sleep EEG recordings in adolescent twins. We hypothesize that the topographic distribution of sleep EEG power will be more similar in monozygotic than dizygotic twins, indicating that regional aspects of the sleep EEG have a genetic component. We also examine the stability of sleep EEG topography within an individual over 6 months.

## Materials and Methods

Fourteen monozygotic (MZ; n = 28; mean age = 13; SD = 1.3; 14 females) and 11 dizygotic (DZ; n = 22; mean age = 13.5; SD = 0.7; 6 females) same-sex twin pairs aged 10 to 15 years participated in the current study. There was no difference between the MZ and DZ group with regards to the distribution of gender (χ^2^ (1,50) = 2.65; p > 0.05) nor age (t (48) = −1.84; p > 0.05). All participants were White with the exception of three twin pairs who were biracial (two pairs White/Asian; one pair Black/White). Pubertal development was assessed by means of a self-rating scale adapted from Petersen *et al*.^49^. According to this scale, all females in our sample were in late or postpubertal stages of development, while the males’ development ranged from mid to postpubertal. Written informed consent was obtained from parents and consent from participants after explaining study procedures in detail. Study procedures were approved by the local ethics committee of the Canton of Zurich and performed according to the Declaration of Helsinki. All participants were healthy and born after the 30th week of pregnancy. Zygosity was determined by means of a questionnaire administered to the parents^50^. This questionnaire is 95 % accurate^50^. Sleep EEG recordings were performed at families’ homes on two consecutive nights at two time points 6 months apart (mean = 195 days; SD = 19 days). Prior to sleep EEG recordings, participants slept on a fixed sleep schedule, ensuring 9.5 to 10 h of sleep per night for at least five days. The first night served as adaptation night, and data from the second (baseline) night was included in the analysis.

Brain activity during sleep was recorded via a Geodesics EEG system (GSN300; Electrical Geodesic Inc., Eugene, OR, USA) with 64-channel nets. Six channels were used for recording of electrooculogram (4 channels) and electromyogram (2 channels), resulting in 58 EEG channels. The data was collected at a sampling rate of 1000 Hz and downsampled to 250 Hz for analysis. The impedance was below 50 kΩ at the start of the recording, which is well within the recommended range for the EGI system^51^. On average, 7 % of channels in a given participant were excluded due to poor signal quality. The signal at each derivation was then recalculated relative to the average of all derivations (average reference).

Data were scored in 30-s epochs according to the criteria of Rechtschaffen and Kales^52^. Power density spectra were calculated per epoch (average of six 5-s windows; Hanning window; no overlap; frequency resolution 0.2 Hz) in MATLAB (Mathworks, Natick MA, USA). Epochs with artifacts were excluded by means of a semi-automated procedure based on power in the low (0.8–4.6 Hz) and high (20–40 Hz) frequencies^13^. Because we include high frequencies (e.g., gamma) in our analysis, and these frequencies are susceptible to artifacts, we performed further artifact correction by careful visual inspection of the time-frequency spectra (spectrograms) to detect noisy segments in the data in addition to examining the topographic distribution of power at high-frequencies to confirm that data were thoroughly cleaned. Because we were interested in topographic distribution independent of absolute power, we normalized the power at each derivation and frequency bin to the total power across all derivations at that frequency bin. This procedure was performed for each subject separately. Within a twin pair the maximal common length of NREMS and REMS (rapid eye movement sleep) epochs was used for analysis. The following frequency bands were examined: delta (1–4.6 Hz), theta (4.8–7.8 Hz), alpha (8–10.8 Hz), sigma (11–16 Hz), beta 1 (16.2–20 Hz), beta 2 (20.2–24 Hz), gamma 1 (24.2–34 Hz) and gamma 2 (34.2–44 Hz).

Pearson correlation coefficients between two vectors (each corresponding to a twin in a pair; i.e., siblings), each vector consisting of normalized power for all derivations (i.e., 58 values corresponding to the number of EEG derivations) were calculated. Thus, for each twin pair, an r-value was obtained resulting in 14 r-values for **MZ** pairs (n = 14 twin pairs) and 11 values for **DZ** twin pairs (n = 11 twin pairs). A third group consisting of pairs that were non-related (i.e., not siblings) was constructed combining individuals from both the MZ and DZ group that were unrelated (e.g., not twin siblings) to each other. Correlation coefficients (r-values) were also computed for this group of subjects (**NR**; n = 990 non-related pairs). Since multiple recordings were available for each subject, Pearson correlation coefficients were also calculated for the same subject between the first and the second assessment (**Self**; assessments separated by 6 months; n = 50). R-values were Fisher’s z-transformed prior to averaging. To assess heritability, we used Falconer’s formula ${h}^{2}=2({r}_{MZ}-{r}_{DZ})$^53^ based on the difference between correlations within MZ (r_MZ_) as compared to DZ (r_DZ_) twin pairs. Shared environmental contributions were estimated according to ${c}^{2}={r}_{MZ}-{h}^{2}={r}_{DZ}-{h}^{2}/2$^54^. In other words, any similarity between identical twins that is not due to genetic factors is necessarily due to environmental factors shared amongst twin pairs.

Fisher’s z -transformed correlation coefficients were subjected to a 5-way ANOVA with between-subject factors **Group** (MZ, DZ, NR) and **Gender** (Female, Male), and within-subject factors **Band** (Delta, Theta, Alpha, Sigma, Beta1, Beta2, Gamma 1, Gamma 2), **State** (NREMS, REMS) and **Time** (Time 1, Time 2). In order to include the Self group in our statistical analyses, we performed a 3-way ANOVA with the between-subject factor **Group** (MZ, DZ, NR, Self) and within-subject factors **Band** (Delta, Theta, Alpha, Sigma, Beta1, Beta 2, Gamma1, Gamma 2) and **State** (NREMS, REMS) including only data from the second assessment (Time 2). All ANOVAs were calculated with the R package *afex* and the post-hoc t-tests with the R package *multcomp*.

In addition, we examined topographic distribution averaged across subjects during NREMS and REMS for 1-Hz frequency bins up to 44 Hz indicated by their upper limits (e.g., the first bin corresponds to the five 0.2-Hz bins centred at 0.2, 0.4, 0.6, 0.8 and 1 Hz), since such data has not previously been published for this age group. In order to assess similarity of the topographic distribution of power between NREMS and REMS, we calculated Pearson correlation coefficients between two vectors, one for NREMS and one for REMS, each consisting of normalized power at all derivations (i.e., 58 values corresponding to 58 derivations). This was done separately for each frequency band.

We also performed a 2-way ANOVA with the between-subject factor **Group** (MZ, DZ) and the within-subject factor **Time** (Time 1, Time 2) on sleep stage variables to test for differences between the groups or changes across time.

The datasets generated and analyzed during the current study are available from the corresponding author on reasonable request and pending ethics approval.

## Results

Sleep stages were as expected for a sample of healthy adolescents in this age group (Table 1). ANOVA analysis revealed no differences between MZ and DZ twins, the two time points or their interaction with regards to any sleep stage parameter.

**Table 1:** Mean and standard deviation (in parentheses) of sleep parameters for monozygotic (MZ; n = 28) and dizygotic (DZ; n = 22) twins at two time points separated by 6 months (mean = 195 days; SD = 19 days).

Sleep Parameter	Time 1		Time 2		ANOVA		
	MZ	DZ	MZ	DZ	Time	Group	Time × Group
Total Sleep Time (min)	526.69 (±56.12)	542.16 (±36.69)	544.98 (±36.22)	528.66 (±26.76)	0.06 (p = 0.81)	0.00 (p = 0.96)	2.61 (p = 0.11)
Wake After Sleep Onset (min)	23.54 (±25.14)	30.41 (±31.22)	19.73 (±17.27)	22.00 (±24.88)	1.21 (p = 0.28)	0.73 (p = 0.40)	0.17 (p = 0.68)
Sleep Latency (min)	22.63 (±19.71)	17.84 (±8.84)	21.73 (±19.20)	19.34 (±8.07)	0.03 (p = 0.87)	0.55 (p = 0.46)	0.43 (p = 0.52)
Sleep Efficiency (%)	91.90 (±4.98)	91.59 (±4.68)	92.90 (±4.92)	92.77 (±4.44)	1.19 (p = 0.28)	0.04 (p = 0.85)	0.01 (p = 0.93)
REMS Latency (min)	109.17 (±45.77)	92.44 (±38.18)	113.23 (±41.87)	101.25 (±49.04)	0.73 (p = 0.40)	1.49 (p = 0.23)	0.10 (p=0.75)
Stage 2 (%)	45.53 (±10.46)	44.53 (±9.31)	43.42 (±9.16)	42.15 (±8.52)	1.15 (p = 0.29)	0.27 (p = 0.61)	0.00 (p = 0.95)
Slow Wave Sleep (%)	29.15 (±9.87)	26.29 (±6.69)	28.57 (±9.13)	29.31 (±10.04)	0.00 (p = 0.99)	0.00 (p = 0.96)	0.09 (p = 0.77)
Stage REMS (%)	25.08 (±5.22)	28.66 (±8.69)	24.63 (±4.29)	25.12 (±4.13)	0.60 (p = 0.44)	0.48 (p = 0.49)	0.12 (p = 0.73)

The percent values were calculated with respect to total sleep time. Sleep latency is defined as the first occurrence of stage 2 sleep following lights out. Results from our 2-way ANOVA with the within-subject factor Time (Time 1, Time 2) and the between-subject factor Group (MZ, DZ) and their interaction are also reported (F-values; p-values in parentheses).

**Figure 1:**
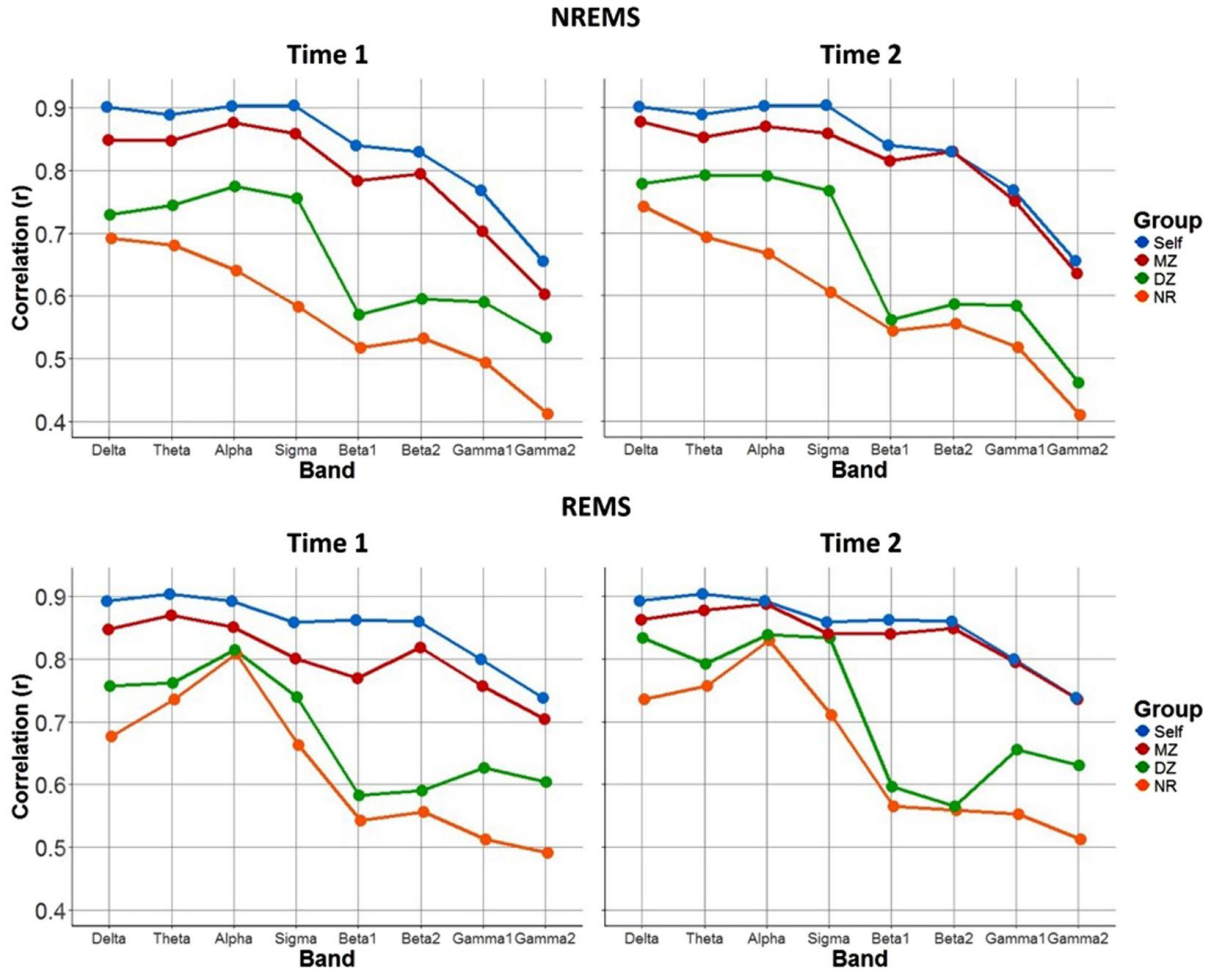
Correlation coefficients averaged for the four groups. Self = two time points in one individual separated by 6 months; MZ = monozygotic twins, DZ = dizygotic twins, NR = non-related individuals. Note that the same data from the Self group is shown in both time 1 and time 2 plots since this measure uses both assessment points. Sample size varied by group (Self n = 50; MZ n = 14; DZ n = 11; NR n = 990).

The Pearson correlation coefficients of normalized power averaged for each of the four groups are shown in Fig. 1 for all frequency bands, the two sleep states and the two time points 6 months apart (mean = 195 days; SD = 19 days). Based on visual inspection, the correlations were highest within an individual (Self: 0.7 ≤ r ≤ 0.9), followed by MZ twin pairs (0.6 ≤ r ≤ 0.9), then DZ twin pairs (0.5 ≤ r ≤ 0.8), and finally NR individuals (0.4 ≤ r ≤ 0.8). This trend was true for both states, both time points and all bands. Generally speaking, we found a trend towards slightly higher correlations at the second assessment (i.e., independent of group; main effect of time p = 0.07), but the distribution of values across bands and states was similar for the two time points.

The degree of correlation in Self, MZ and DZ groups was similar across bands (delta to sigma; Fig. 1). NR correlations, however, manifested greater frequency and state dependent modulation (Fig. 1). For the NR group in both NREMS and REMS, a declining trend of correlation coefficients can be seen from delta (r = 0.74; time 2) to sigma (r = 0.61; time 2) during NREMS. Conversely, during REMS highest correlations were observed for alpha (r = 0.83; time 2). In the two beta (i.e., beta 1 and beta 2) and the two gamma (i.e., gamma 1 and gamma 2) bands, we found lower correlations for all subject groups in both sleep states.

**Figure 2:**
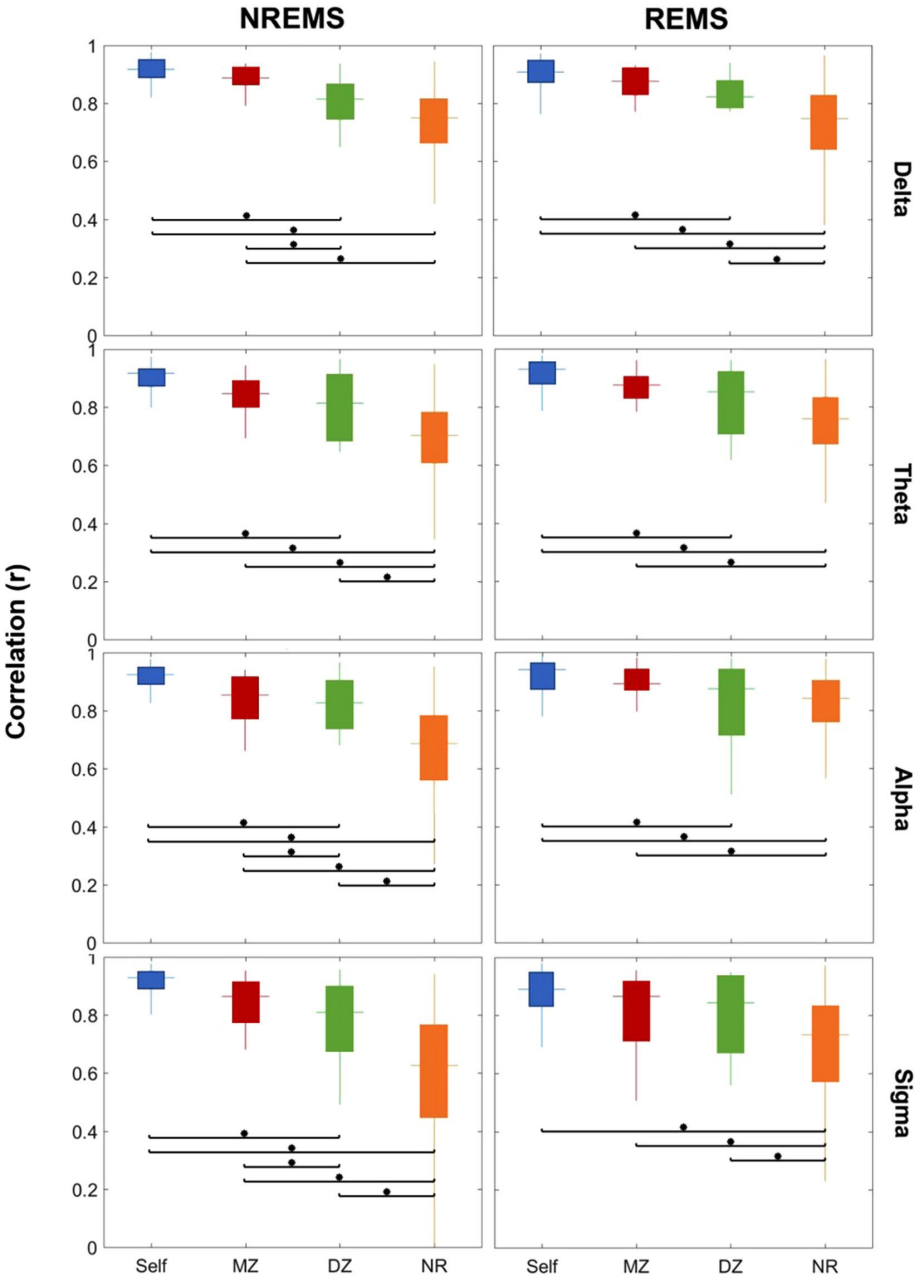
Box plots of correlation coefficients within each group, for the frequency bands delta, theta, alpha and sigma, and the two states, NREMS and REMS. The bottom and top edges of each box indicate the first and third quartiles, the central line indicates the median, and the maximum whisker length is defined as 1.5 times the interquartile range, ending at the maximum value in this range. Differences between the groups were evaluated by means of Tukey-corrected post-hoc t-tests on Fisher’s z-transformed values. The significant differences (p < 0.05) are depicted with an asterisk.

**Figure 3:**
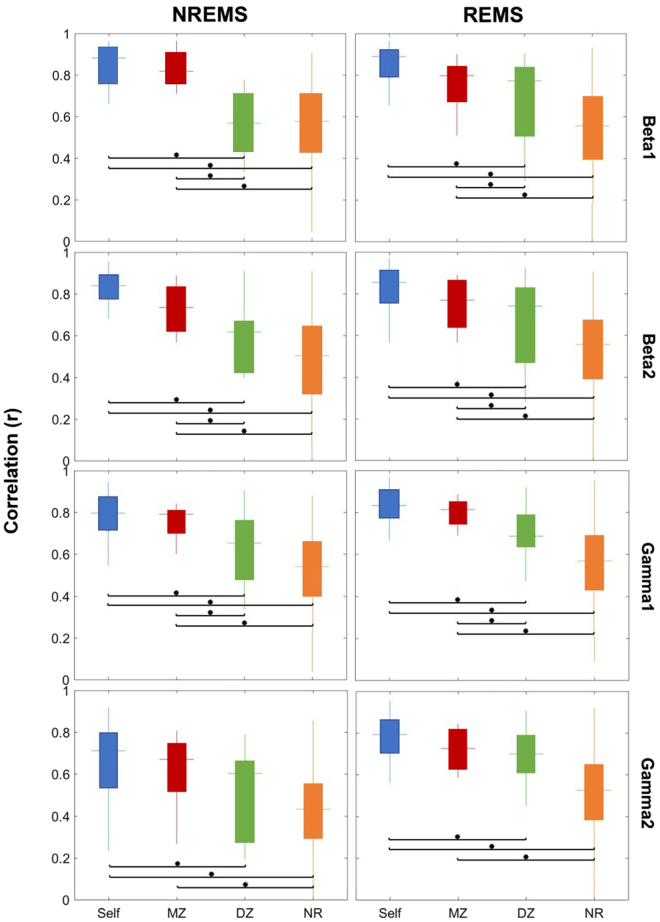
Box plots of correlation coefficients within each group, for the frequency bands beta 1, beta 2, gamma 1 and gamma 2, and the two states, NREMS and REMS. The bottom and top edges of each box indicate the first and third quartiles, the central line indicates the median, and the maximum whisker length is defined as 1.5 times the interquartile range, ending at the maximum value in this range. Differences between the groups were evaluated by means of Tukey-corrected post-hoc t-tests on Fisher’s z-transformed values. The significant differences (p < 0.05) are depicted with an asterisk.

Since the factors **Time** (p = 0.07) and **Gender** (p = 0.48) were not significant and had no significant interactions with the other factors, we focused on the second assessment for further analyses and did not include Gender as a factor. ANOVA results for the second time point with the three factors (Group, Band and State) and their interactions are shown in Table 2. All three factors and their interactions were significant, except for the interaction between Group and State which remains a trend (p = 0.1). Differences between the four groups were dependent on the sleep state and the frequency band (Figs 2 and 3; post-hoc t-tests).

**Table 2:** A 3-way ANOVA was performed on Fisher’s z-transformed correlation coefficients with the between-subject factor Group (Monozygotic, Dizygotic, Non-Related, Self) and within-subject factors Band (Delta, Theta, Alpha, Sigma, Beta 1, Beta 2, Gamma 1, Gamma 2) and State (NREMS, REMS).

Effect	df	F	p-value
Group	1	213.95	<0.0001
Band	7	118.61	<0.0001
State	1	31.89	<0.0001
Group x Band	7	5.61	<0.0001
Group x State	1	2.09	0.10
Band x State	7	4.74	0.0006
Group x Band x State	7	6.60	<0.0001

The factors Group, Band and State were significant, as well as all their interactions with the exception of Group x State. The total number of degrees of freedom (df) was 49.

**Figure 4:**
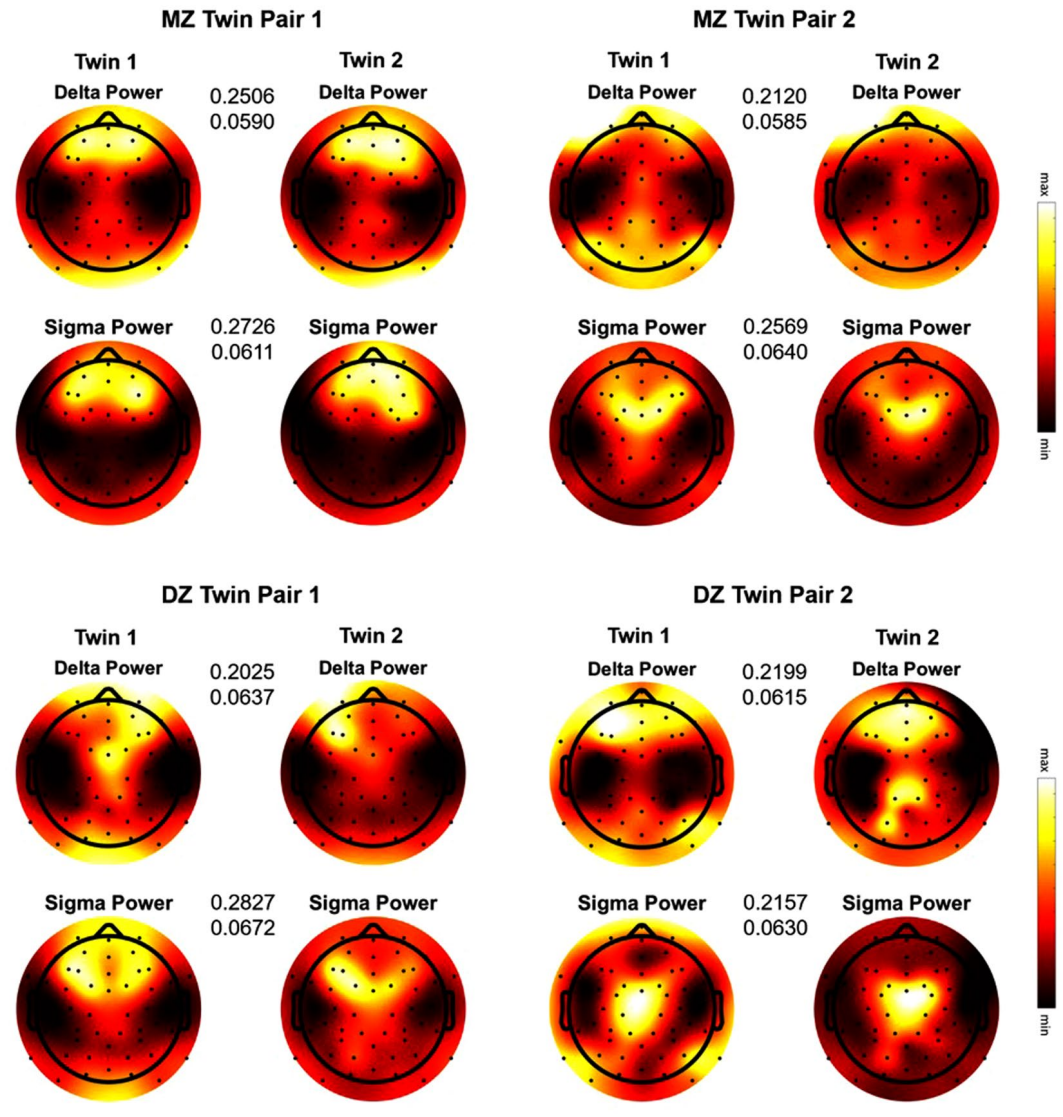
Topographic distribution of normalized EEG power during NREMS for two exemplary MZ and DZ pairs. Greater similarity in the pattern within the MZ pairs as compared to the DZ pairs is visually apparent. The maps were scaled within a twin pair for each frequency band separately.

In general, topographic similarity was largest for the Self and MZ group as compared to the other groups as reflected in significantly higher correlations (Figs 2 and 3) and no statistically significant difference between the Self and MZ group. The difference between MZ and DZ twin pairs and its significance varied by frequency band and sleep state (from p <0.0001 in beta 1 and beta 2 bands during both sleep states to p = 0.9 in delta and sigma bands during REMS). For NREMS the difference between MZ and DZ was significant for all bands except for a trend in the theta (p = 0.2) and gamma2 (p = 0.1) bands. To demonstrate the topographic similarity of MZ twins as compared to DZ twins visually, topographic maps for two exemplary MZ and DZ pairs in the delta and sigma bands during NREMS are shown in Fig. 4. On the other hand, for REMS we observed significant differences between MZ and DZ twins for beta1, beta2, and gamma1 and a trend for theta (p = 0.1). Despite this, we note that the observed average correlation was qualitatively higher for MZ as compared to DZ and, MZ twins showed a narrower range of correlation coefficients as reflected in the distribution of values (Figs 2 and 3).

**Figure 5:**
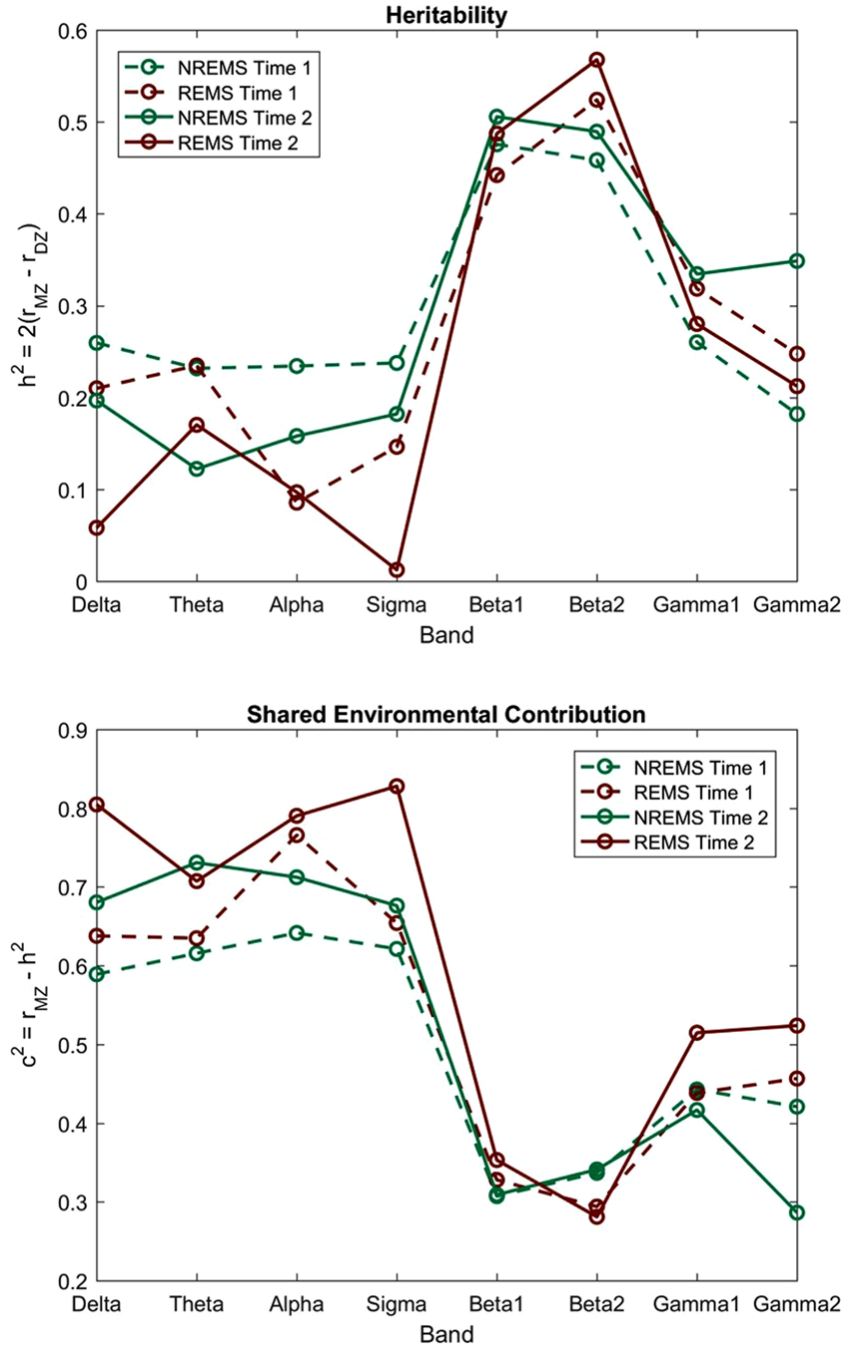
Heritability estimated by means of Falconer’s formula ${h}^{2}=2({r}_{MZ}-{r}_{DZ})$ and shared environmental contributions calculated from ${c}^{2}={r}_{MZ}-{h}^{2}={r}_{DZ}-{h}^{2}/2$ for NREMS and REMS. Data from the first assessment (time 1) are shown as a dashed line and data from the second assessment (time 2) as a solid line. REMS is shown in maroon while NREMS is in teal.

Figure 5 depicts our heritability estimates (h^2^) based on Falconer’s formula and shows that heritability depends on frequency band and the sleep state, supporting our previous results. Apart from small changes in heritability, the overall distribution of values across bands remained stable between the two time points. The maxima were observed for beta 1 during NREMS (h^2^ = 0.48 at time 1 and h^2^ = 0.51 at time 2) and for beta 2 during REMS (h^2^ = 0.52 at time 1 and h^2^ = 0.57 at time 2). Figure 5 also shows the corresponding estimates for shared environmental contributions (c^2^), which were high for delta to sigma bands and low in the other bands.

**Figure 6:**
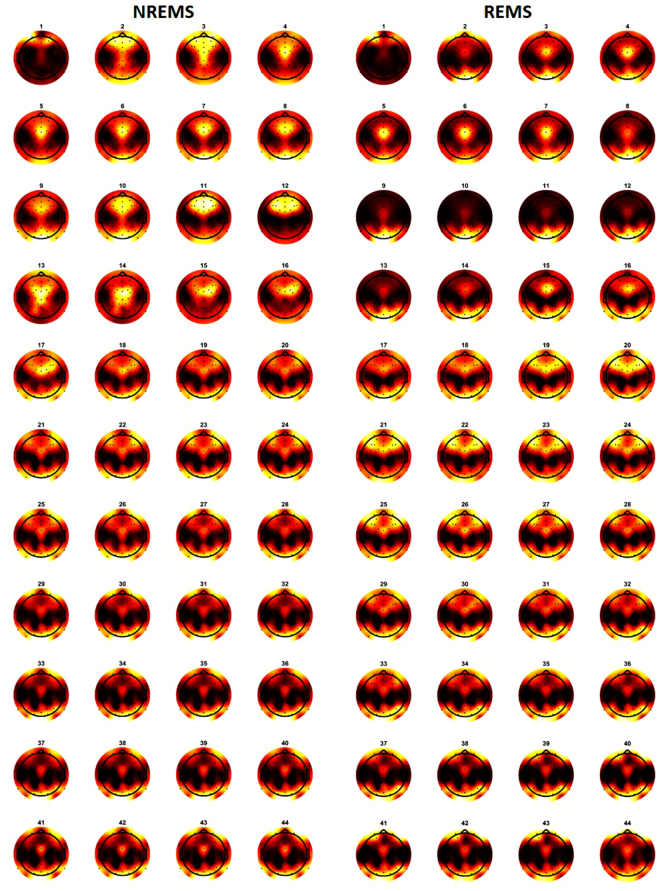
Average (n = 50) topographic distribution of EEG power normalized to the total power during NREMS and REMS for 1 Hz bins up to 44 Hz. The numbers indicate the upper limit of the frequency bin (e.g., 1 corresponds to the five 0.2-Hz bins centered at 0.2, 0.4, 0.6, 0.8 and 1 Hz). Each map was scaled separately between its minimal and its maximal value.

Since we found a different magnitude of correlation coefficients for NREMS and REMS, particularly with regards to the NR group, we examined the topographic similarity between NREMS and REMS for each frequency band across all subjects (Table 3). We found high and significant correlations (>0.8) between the two sleep states in theta, beta 1, beta 2, gamma 1 and gamma 2 bands. Delta and alpha were in the same range with statistically significant correlation coefficients of 0.68 and 0.62 respectively. Sigma, on the other hand, manifested the lowest correlation of 0.19 (p = 0.26). Figure 6 shows the topographic distributions of normalized EEG power during NREMS and REMS for 1 Hz bins up to 44 Hz averaged across subjects.

**Table 3:** Correlation coefficients between NREMS and REMS topographic distributions averaged for the eight frequency bands and the corresponding p-values.

Band	r	p-value
Delta	0.6763	<0.0001
Theta	0.8412	<0.0001
Alpha	0.6170	0.02
Sigma	0.1871	0.26
Beta 1	0.8870	<0.0001
Beta 2	0.8705	0.006
Gamma 1	0.8762	0.003
Gamma 2	0.7993	0.002

## Discussion

### Sleep EEG Topography as a Complex Endophenotype

In this study, we used a twin design in order to evaluate the degree to which genes contribute to the topographic distribution of sleep EEG power in adolescents. The estimation of heritability is based on the assumption that greater similarity between MZ as compared to DZ twins can be attributed to the larger proportion of shared genes amongst MZ twins, while high and significant associations between both MZ and DZ twins imply an environmental impact. We found significant genetic influence on sleep EEG topography during NREMS, as reflected in significant differences between MZ and DZ twin pairs. This difference between MZ and DZ pairs was largely absent in REMS with the exception of beta 1, beta 2 and gamma 1 bands.

Despite the significant differences for multiple bands in NREMS, heritability was low (0.12 ≤ h^2^ ≤ 0.2 for NREMS and 0.01 ≤ h^2^ ≤ 0.2 for REMS) in delta to sigma bands in contrast to what has been reported for absolute power in twin studies of the sleep EEG in adults (h^2^ = 0.96 for 8 to 16 Hz power spectra^37^). We hypothesize that this is because absolute power reflects brain structure^22,55,56^ while the topographic distribution of power is more indicative of brain networks. On the other hand, we found higher heritability estimates in the beta bands (0.44 ≤ h^2^ ≤ 0.57) for both NREMS and REMS sleep and in a range similar to heritability reported for psychiatric disorders^57^. High frequency ranges are often overlooked with regards to sleep EEG analyses in healthy populations, largely due to the difficulties in dealing with artifacts found at these frequencies. However, we went through great effort to clean our data and have ruled out a significant impact of artifacts on our findings based on (1) high topographic stability within an individual across six months and (2) high similarity between twin pairs. Beta activity in waking has been linked to a number of cognitive functions^58–60^, including the selection of relevant stimuli^61,62^. Furthermore, abnormal beta activity during waking has been observed in several neurological disorders, such as schizophrenia^61,63^, Alzheimer’s disease^64^, attention deficit hyperactivity disorder^65^, post-traumatic stress disorder^66^, panic disorder^67^ and substance use disorders^68,69^. Based on these findings and the high genetic impact on beta ***power*** (heritability of 85 %) in the ***waking*** EEG as revealed by twin studies^70,71^, beta band EEG ***power*** has been proposed as a potential endophenotype for several psychiatric disorders^72,73^. With regards to the sleep EEG, insomnia in adolescents and adults is associated with elevated beta ***power*** in NREMS and has been proposed as an indicator of hyperarousal and thus an informative metric in sleep^74,75^. We suggest that by combining power with topography an even stronger endophenotype may evolve. Further adding to the utility of the sleep EEG as an endophenotype, is that during sleep the influence of external (e.g., experimenter presence) and internal (e.g., attention) factors is minimized.

In contrast to beta power in NREMS and REMS, we find evidence of a strong shared environmental influence on EEG topography in all other frequency bands (delta, theta, alpha, sigma and gamma 2), as evidenced by high correlations for both MZ and DZ twin pairs. The specific environmental factors affecting such topography remain unknown, however, previous studies have identified a wide range of environmental factors affecting brain development. These include, but are not limited to child-parent interactions, stress, and socio-economic status^76,77^. How these factors influence sleep EEG topography should be examined in future studies. Nonetheless, in line with our findings, functional magnetic resonance imagining (fMRI) studies in twins have shown that resting state networks in waking are largely driven by environmental factors^78^, suggesting that brain networks have a strong environmental component.

### Topographic Distribution of EEG Power during NREMS and REMS in Adolescence

The topographic distributions of NREMS EEG power in our study were similar to those seen for this age group by Kurth *et al*.^10^. To our knowledge, this is the first paper to use high-density sleep EEG to show the topographic distribution of REMS EEG power in an adolescent sample. Visual comparison of our REMS topographic maps to those published for adults^9^ reveals globally similar patterns. When comparing the topographies between the two states, NREMS and REMS, we found pronounced similarities, as reflected in high and significant correlation coefficients. The highest correlations were observed for the theta, beta and gamma bands, while correlation coefficients were smallest in the alpha and sigma bands. These findings may be due to similar neuronal circuitry underlying the two states in theta, beta and gamma bands, while oscillations unique to each state (e.g., spindles in NREMS) might explain the low correlations in alpha and sigma bands.

In addition, we found high temporal stability of regional power distribution within an individual in both NREMS and REMS. Several studies have shown temporal stability of EEG topography during waking^79–81^ and sleep^9,17,18^. However, these studies compared data that was at most a few weeks apart, which is a shorter time interval as compared to the 6 months in our study. Our results indicate that sleep EEG topography may be a trait that remains stable and unique to an individual over longer time periods despite neurodevelopment.

### Associations between Non-Related Individuals

As compared to the other subject groups, non-related individuals exhibited low correlations in sigma during NREMS at both time points (0.58 ≤ r ≤ 0.60). Sigma oscillations during NREMS are generated by thalamo-cortical circuits and have been associated with synaptic plasticity and memory consolidation^82–84^. Furthermore, reduced activity in this frequency range over centro-parietal regions is associated with schizophrenia^40,42,85^. Thus, given the clinical relevance of sigma power, combined with the large inter-individual variability identified in our study, sigma topography might be a useful marker in adolescence for identifying markers of vulnerability to neuropsychiatric and neurological illnesses or subgroups within a diagnostic category (e.g., medication responders versus non-responders). Future studies should consider defining regions of interest on an individual level based on regions that show maximal/minimal power.

### Limitations

Due to the nature of our research question, we were only able to apply traditional heritability analysis (i.e., Falconer’s formula) that has some inherent limitations^86^. Furthermore, the interval of 6 months between the two assessments may be too short to detect developmental effects. Given the significant topographic shifts in the location of maximal power across adolescence^10^, future studies over longer time periods are necessary to determine if the genetic contribution to sleep EEG topography is stable across adolescence. Finally, our sample size was modest and may account for some of the non-significant trends observed between MZ and DZ individuals for REMS. Moreover, the absence of gender effects on heritability may be due to the small sample size and should be addressed in future studies. Nonetheless, we show that sleep EEG topography at higher frequencies is highly heritable, while at lower frequencies shared environmental factors play a more prominent role.

## Conclusion

Sleep EEG topography varies by frequency band and sleep state, but is stable within an individual across time. The observed frequency and state dependence implies that the topographic distribution of power is not only a reflection of brain anatomy, but possibly also its underlying brain function. We observed heritability estimates from 1 % to 57 % depending on frequency band and sleep state, which is similar to heritability estimates of brain function (≈40 %) but lower than those reported for brain structure (60–80 %)^87^. Therefore, brain topography likely primarily reflects brain function and thus might be an overlooked, yet potentially critical aspect of brain networks. Our study also suggests that the incorporation of topography into studies looking for a biomarker (e.g., a stable metric that is also genetically determined) of psychiatric and neurodevelopmental disorders may be fruitful.
